# Development of a stress-induced mutagenesis module for autonomous adaptive evolution of *Escherichia coli* to improve its stress tolerance

**DOI:** 10.1186/s13068-015-0276-1

**Published:** 2015-06-26

**Authors:** Linjiang Zhu, Yin Li, Zhen Cai

**Affiliations:** CAS Key Laboratory of Microbial Physiological and Metabolic Engineering, Institute of Microbiology, Chinese Academy of Sciences, No. 1 West Beichen Road, Chaoyang District, Beijing, 100101 China; Key Laboratory of Industrial Biotechnology, Ministry of Education of China, School of Biotechnology, Jiangnan University, Wuxi, 214122 China

**Keywords:** Adaptive evolution, Stress-induced mutagenesis, Genetic toggle switch, Synthetic evolution module, Stress tolerance, *n*-butanol

## Abstract

**Background:**

Microbial tolerance to different environmental stresses is of importance for efficient production of biofuels and biochemical. Such traits are often improved by evolutionary engineering approaches including mutagen-induced mutagenesis and successive passage. In contrast to these approaches which generate mutations in rapidly growing cells, recent research showed that mutations could be generated in non-dividing cells under stressful but non-lethal conditions, leading to the birth of the theory of stress-induced mutagenesis (SIM). A molecular mechanism of SIM has been elucidated to be mutagenic repair of DNA breaks. This inspired us to develop a synthetic SIM module to simulate the mutagenic cellular response so as to accelerate microbial adaptive evolution for an improved stress tolerance.

**Results:**

A controllable SIM evolution module was devised based on a genetic toggle switch in *Escherichia coli*. The synthetic module enables expression and repression of the genes related to up- and down-regulation responses during SIM in a bistable way. Upon addition of different inducers, the module can be turned on or off, triggering transition to a mutagenic or a high-fidelity state and thus allowing periodic adaptive evolution. Six genes (*recA*, *dinB*, *umuD*, *ropS*, *ropE*, and *nusA*) in the up-regulation responses were evaluated for their potentials to enhance the SIM rate. Expression of *recA*, *dinB*, or *ropS* alone increased the SIM rate by 4.5- to 13.7-fold, whereas their combined expression improved the rate by 31.9-fold. Besides, deletion of *mutL* increased the SIM rate by 82-fold. Assembly of these genes into the SIM module in the *mutL*-deletion *E. coli* strain elevated the SIM rate by nearly 3000-fold. Accelerated adaptive evolution of *E. coli* equipped with this synthetic SIM module was demonstrated under *n*-butanol stress, with the minimal inhibitory concentration of *n*-butanol increasing by 56 % within 2.5 months.

**Conclusions:**

A synthetic SIM module was constructed to simulate cellular mutagenic responses during SIM. Based on this, a novel evolutionary engineering approach—SIM-based adaptive evolution—was developed to improve the *n*-butanol tolerance of *E. coli*.

**Electronic supplementary material:**

The online version of this article (doi:10.1186/s13068-015-0276-1) contains supplementary material, which is available to authorized users.

## Background

The past decade has witnessed the power of synthetic biology in the microbial production of fuels and chemicals from renewable resources. Much effort has been directed towards the design and development of novel synthetic genes, pathways, circuits, and modules to improve productivity [1–4]. However, few studies have concentrated on the stress tolerance of microbes, an important requirement for an efficient industrial production process [5, 6]. We have considered whether microbial stress tolerance, which is a complex physiological response to environmental perturbation [7], could be effectively engineered using the powerful concepts and tools of synthetic biology [6].

We aimed to improve microbial stress tolerance by endowing cells with the capability of autonomous adaptive evolution when facing environmental stresses. This can be achieved by equipping cells with a synthetic evolution module. The evolution module was designed to increase the cellular mutation rate in response to stress signals, based on a new evolutionary theory of stress-induced mutagenesis (SIM). SIM experiments indicate that non-lethal stress will inhibit cell growth and trigger intracellular state transition to a transient hypermutation state in a small subpopulation (<0.1 %) of cells, thereby promoting adaptive evolution under stressful condition [8–10]. This inspired us to construct a SIM-based adaptive evolution module to regulate the hypermutation state for autonomous adaptive evolution under stresses.

SIM is believed to be associated with various cellular network responses of mutagenic DNA repair, including up-regulation of the SOS response, general stress response (RopS response), envelope stress response (RopE or σ^E^ response), and heat-shock response, and down-regulation of mismatch repair (MMR) [9–13]. Therefore, the synthetic SIM module was required to control up-regulation and down-regulation responses simultaneously. Although more than 93 genes are involved in the network of SIM, we only selected six candidate genes (*recA*, *dinB*, *umuD’*, *ropE*, *ropS*, and *nusA*) related to up-regulation responses and determined the effects of their individual and combined expression on SIM rates. Among them, the *recA*, *ropS*, and *ropE* genes encode the main acting proteins during the above three SOS, RopS, and RopE responses, the *dinB* and *umuD’* are the encoding genes for the main error-prone DNA polymerases Pol IV and Pol V, and the *nusA* gene encodes the transcription elongation factor NusA. The genes that promoted SIM upon expression, together with the *mutL* gene, which elevated SIM rate after deletion [14], were assembled into a synthetic toggle switch to construct the SIM module. After introducing the module into a previously constructed *mutL-*deficient *E. coli* strain [14], transition to a mutagenic or high-fidelity cellular state could be conducted by turning the module on or off via addition of different inducers. Finally, the evolution module was applied to produce autonomous adaptive evolution of *E. coli* under *n*-butanol stress.

## Results

### Design of the SIM module

**Fig. 1:**
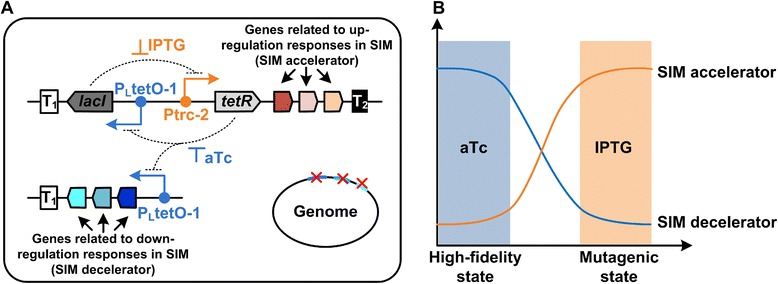
The design (**a**) and regulation (**b**) of the SIM module

SIM is the consequence of up-regulation of SOS, RopS, and RopE responses and down-regulation of MMR under stress. All responses prompt DNA replication in an error-prone manner and consequently result in a transient mutagenic state, with mutation rates increased by several orders of magnitude [8–10]. The genes related to up-regulation and down-regulation responses can be termed as SIM accelerators and decelerators, respectively. Therefore, the SIM module was designed to control the expression and repression of “SIM accelerator” and “SIM decelerator” genes in a bistable way (Fig. 1). A synthetic toggle switch, composed of two inducible promoters, Ptrc-2 and P_L_tetO-1, and their corresponding repressors, LacI and TetR [15], was employed to implement the bistable control. The genes responsible for accelerating SIM were placed under the control of the isopropyl-β-D-1-thiogalactopyranoside (IPTG)-induced Ptrc-2 promoter. The genes related to repress SIM were first knocked out and then relocated downstream of the anhydrotetracycline (aTc)-induced P_L_tetO-1 promoter. Theoretically, when IPTG is added, the SIM accelerator genes are over-expressed and the SIM decelerator genes are repressed, resulting in a high mutation rate during genome replication (the mutagenic state). Upon aTc addition, cells switch back to the high-fidelity state because the SIM decelerator genes are over-expressed and the SIM accelerator genes are repressed.

When the green fluorescent protein (GFP) gene was separately inserted into the two control units of the toggle switch, its expression/repression pattern strictly and quickly responded to the addition of the two inducers, confirming the bistability of the synthetic toggle switch in regulation of gene expression/repression (Additional file 1: Figure S1).

### Component selection for the SIM module

The main down-regulation response occurs in the MMR system, essential proteins of which are MutL, MutS, and MutH. Our previous work indicated that deletion of MutL significantly increased the SIM rate, indicating an important role of MutL in SIM [14]. Hence, the MutL encoding gene, *mutL*, was selected as an SIM decelerator that needed to be down-regulated. This was achieved by placing it under the control of the P_L_tetO-1 promoter in the plasmid pML, which was derived from pACYC184 (Additional file 1: Figure S2D).

**Fig. 2:**
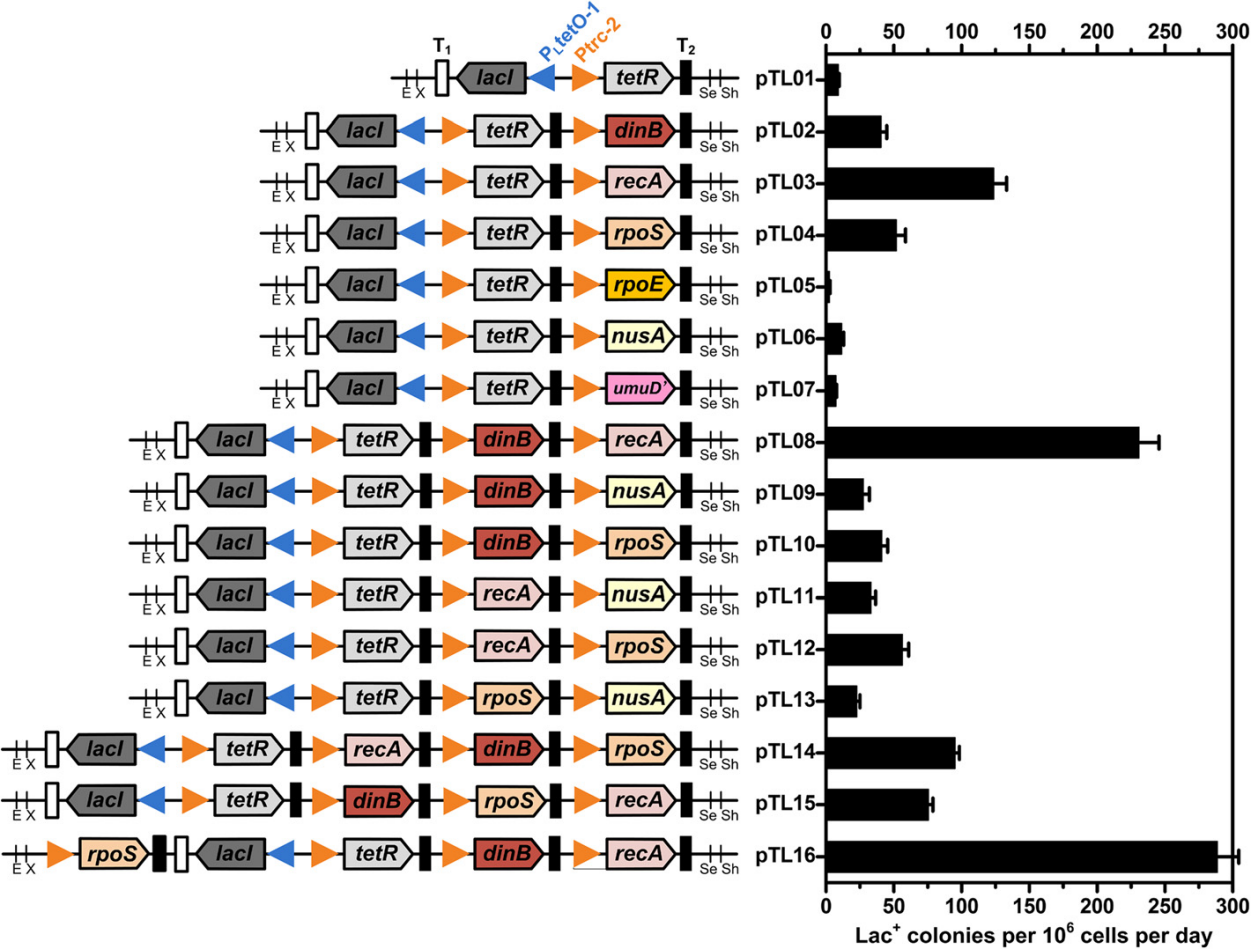
The genetic construction of plasmids pTL01-16 and the frequency of Lac^+^ colonies at day 6 for *E. coli* FC40 strains harboring them. *E Eco*RI, *X Xba*I, *Se Spe*I, *Sh Sph*I

For the up-regulation responses which include SOS, RopS, and RopE, a network of more than 93 genes has been identified by the decrease of SIM rate after transposon-induced deactivation of each gene [13]. However, their effects on elevating the SIM rate by expression are still unknown. In this work, six genes encoding the main acting proteins RecA (*recA*), RopS (*ropS*), and RopE (*ropE*) in the three up-regulation responses, the main error-prone DNA polymerase Pol IV (*dinB*) and Pol V (*umuD’*), and the transcription elongation factor NusA (*nusA*) were selected to test their SIM rates after expression. The genes along with appropriate ribosomal binding site sequences (Additional file 1: Figure S2B) and the modified BioBrick enzyme connections [16], were first cloned into the *Xba*I/*Sph*I sites of pUC19 (Additional file 1: Figure S2A). The six *Xba*I/*Sph*I digested gene units were then separately inserted into the *Spe*I/*Sph*I digested pTL01 (Additional file 1: Figure S2C), yielding pTL02-07, each containing a single gene (Fig. 2). Integration of another one or two gene units into pTL02-07 was similarly conducted by BioBrick assembly, resulting in the two- or three-gene-containing pTL08-16 (Fig. 2).

All pTL plasmids were transformed into *E. coli* FC40 to determine their SIM rates under lactose starvation. *E. coli* FC40 [RifR, F’ (*lacIΩZ*), *ara*, Δ(*lacproB*), *thi*], which is the model strain for quantitative SIM rate assay, constitutively expresses a *lacI-lacZ* fusion containing a +1 frameshift mutation on the F’ conjugative plasmid that renders it Lac^−^ [17]. The rapid appearance of Lac^+^ revertants on lactose plates after incubation of 2 days is thought to be derived from spontaneous mutation during cultivation, whereas the gradual appearance of Lac^+^ revertants during prolonged incubation (3–6 days) is believed to be the consequence of SIM under lactose starvation [17]. The rate of SIM is represented by the increase rate of the total number of Lac^+^ reversion within 3–6 days, which can be calculated by the total Lac^+^ colonies on day 6 minus those appeared on day 2 and then divided by 4 days [13]. For the single-gene plasmids, to our surprise, only pTL02 (*dinB*), pTL03 (*recA*), pTL04 (*rpoS*), and pTL06 (*nusA*) increased the SIM rate by 4.5-, 13.7-, 5.7-, and 1.3-fold, respectively, compared with the control plasmid pTL01 (Fig. 2, Additional file 1: Table S3). Two combinations of the four genes in plasmids pTL08-13 increased the SIM rate by 2.5- to 25.5-fold. However, the combination of *nusA* with each of the other three genes reduced the SIM rate (pTL09, pTL11, and pTL13 compared with pTL02, pTL03, and pTL04, respectively). Therefore, only *dinB*, *recA*, and *rpoS* were selected to be SIM accelerators that needed to be up-regulated. Further gene-order optimization of these genes in pTL16 produced a highest SIM rate that was 31.9-fold greater than that of the control strain, *E. coli* FC40/pTL01.

### Assembly of the SIM module

**Fig. 3:**
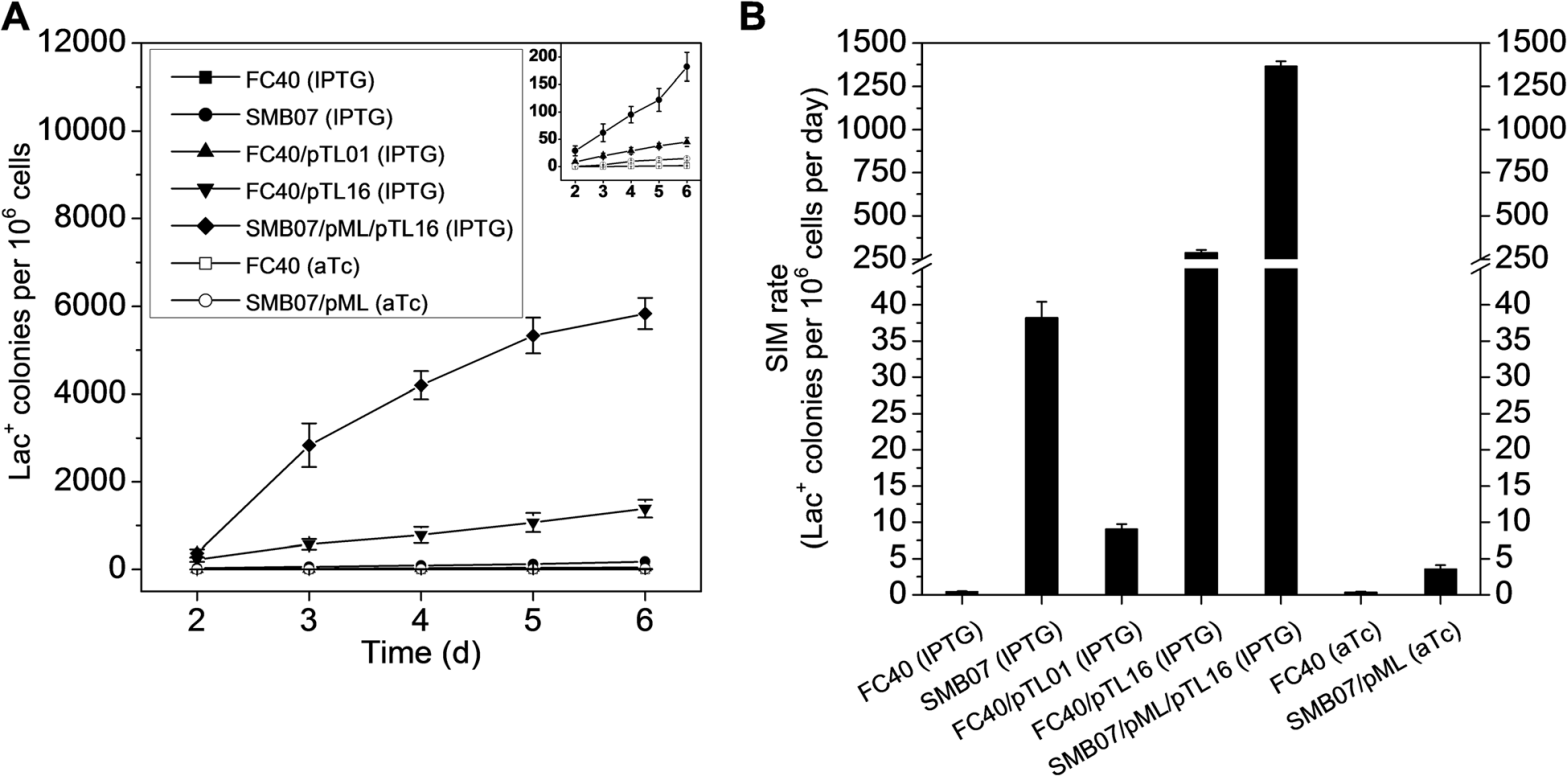
The Lac^+^ reversion mutation rate of strains *FC40*, *SMB07*, *FC40*/*pTL01*, *FC40*/*pTL16*, *SMB07*/*pML*/*pTL16*, and *SMB07*/*pML* in the presence of different inducers. An enlarged graph of the curves of the first three and the last two strains is shown in the upper right corner

As depicted in Fig. 1, the SIM module was composed of two plasmids under the control of the toggle switch: pTL16 regulated the SIM accelerator genes, *dinB*, *recA*, and *rpoS*, while pML controlled the SIM decelerator gene, *mutL*. This SIM module was transformed into SMB07 (a *mutL*-deleted mutant strain of *E. coli* FC40 [14]), yielding the recombinant strain, SMB07/pML/pTL16. This strain, together with FC40/pTL01, FC40/pTL16, SMB07, and FC40, was subjected to the Lac^+^ reversion mutation assay in the presence of IPTG to evaluate their SIM rates (Fig. 3, Additional file 1: Table S4).

For all strains, the number of Lac^+^ colonies increased almost linearly within 3–6 days of incubation with IPTG, showing a typical SIM profile. The FC40 strain showed a SIM rate of 0.4–0.5 Lac^+^ colonies per 10^6^ cells per day in the presence of IPTG or aTc, which was comparable with the reported SIM rate of an independently constructed FC40 strain with *araBAD* deletion (0.5 Lac^+^ colonies per 10^6^ cells per day) [13]. Compared with the original FC40 strain, disruption of the *mutL* gene in SMB07 increased the SIM rate by 82-fold. Using the same control strain FC40, up-regulation of the genes *dinB*, *recA*, and *rpoS* in FC40/pTL16 elevated the SIM rate by 618-fold, although the empty pTL01 plasmid alone raised the SIM rate by 19-fold. However, the combination of *mutL* disruption and *dinB*-*recA*-*rpoS* expression in SMB07/pML/pTL16 only resulted in a 2923-fold increased SIM rate over FC40, which was much lower than the product of the individual increases contributed by the two parts (82-fold times 618-fold). One possible reason for this is the leaky expression of the P_L_tetO-1 promoter. As shown in Additional file 1: Figure S1D and F, a low level of fluorescence was detected for the strain containing the P_L_tetO-1-controlled *gfp* in medium without any inducer. Another possible reason might be the addition of 25 μg/mL of tetracycline to maintain the plasmid pML. Because of its similarity to aTc, tetracycline at this concentration might also serve as a weak inducer. Therefore, the expression of *mutL* and the repression of *dinB*-*recA*-*rpoS* might be induced to a certain degree, both of which restricted the increase of SIM rate.

The results above clearly showed that IPTG turned on the SIM module and switched the cell to the mutagenic state. However, during the Lac^+^ reversion mutation assay, the SIM module cannot be turned off to achieve the high-fidelity state, since lactose can induce the “SIM accelerator” genes under the control of the Ptrc-2 promoter in pTL16. To test whether aTc can turn off the module and switch the cell back to the high-fidelity state, the module was dissected into the “SIM decelerator” part (SMB07/pML) and the “SIM accelerator” part (pTL16), and the function of aTc on each part was separately tested.

Although the SIM rate of strain SMB07/pML in the presence of aTc was a little higher than that of FC40, the former value was much lower than those of IPTG-induced SMB07 and SMB07/pML/pTL16 (Fig. 3, Additional file 1: Table S4), demonstrating that aTc successfully activated the “SIM decelerator” part and reduced the SIM rate to a basal level. The function of aTc in deactivating the “SIM accelerator” part was demonstrated by its ability to repress the expression of “SIM accelerator” genes. To give a quantitative result, the “SIM accelerator” genes in pTL16 were replaced by the *gfp* gene to construct plasmid pTLCG. The fluorescence of the aTc-induced strain SMB07/pML/pTLCG was less than 2 % of the IPTG-induced same strain (Additional file 1: Figure S3). It can be speculated that aTc can also significantly reduce the expression of the “SIM accelerator” genes in pTL16 and restrict the SIM rate to the basal level. Although the absolute SIM rate of the SIM module in the presence of aTc was unable to be represented, the above results clearly showed that aTc was able to restrict the SIM rate at a basal level and switch the cell to the high-fidelity state.

### Improving *n*-butanol tolerance of *E. coli* with the SIM module

**Fig. 4:**
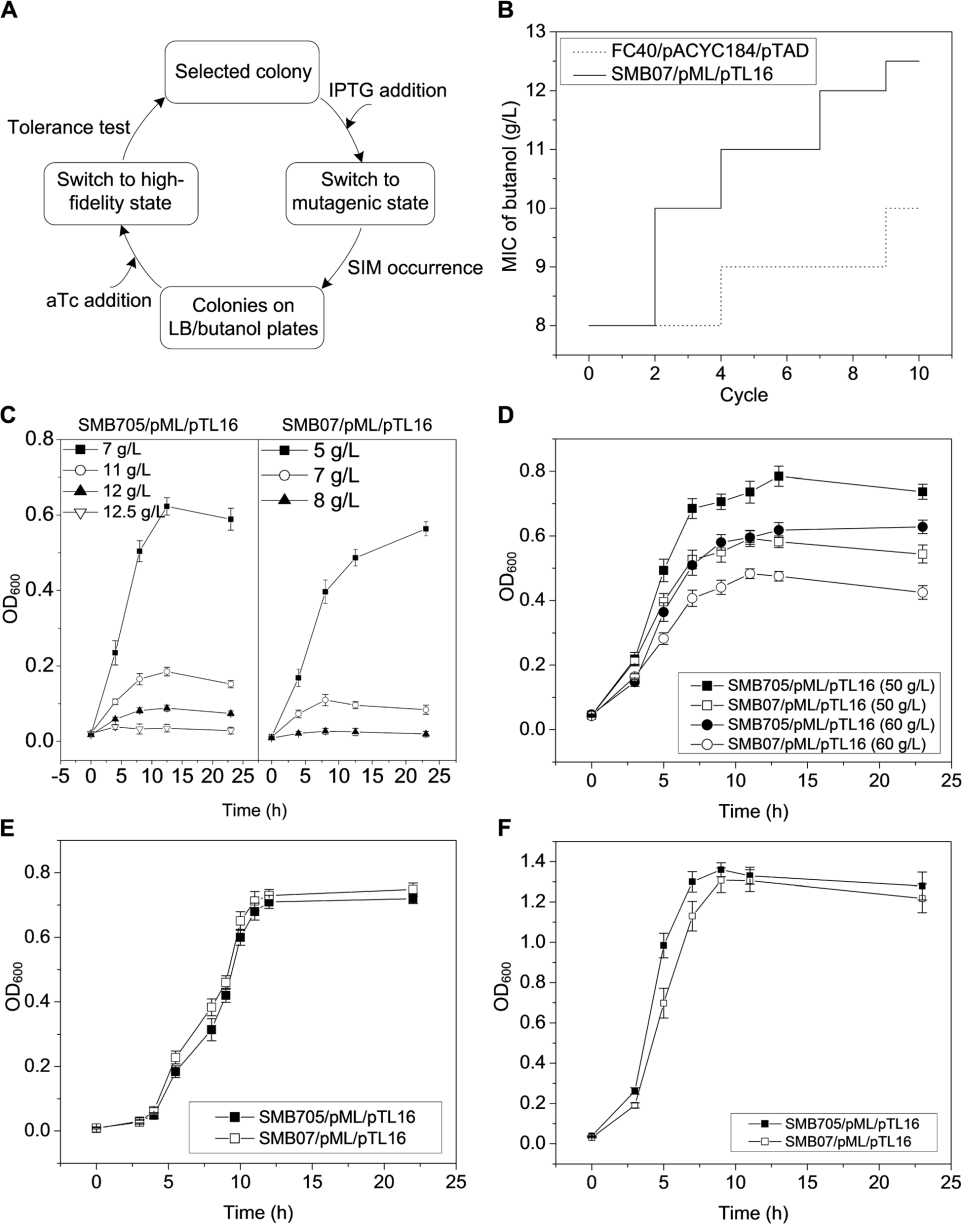
Improving *n*-butanol tolerance of *E. coli* by periodic adaptive evolution via the SIM module. **a** The scheme. **b** The *n*-butanol MIC after each cycle of evolution. The strain *SMB07*/*pML*/*pTL16* equipped with the SIM module and the control strain *FC40*/*pACYC184*/*pTAD* without the module were compared. **c-f** Growth curves of the evolved strain *SMB705*/*pML*/*pTL16* and its parent strain *SMB07*/*pML*/*pTL16* at 37 °C in various media: **c** LB medium with various concentrations of *n*-butanol; **d** LB medium with 50 or 60 g/L NaCl; **e** LB medium at pH 4.8, adjusted by lactic acid; **f** M9 minimal medium containing 0.2 % glucose. For all the media, 100 μg/mL ampicillin, 25 μg/mL tetracycline, and 500 ng/mL aTc were added. All the OD_600_ values were detected using 200 μL of culture in 96-well microplates. Three independent cultures were prepared for each condition

*n*-Butanol is an important renewable biofuel that has attracted much attention [18]. Metabolic engineering has enabled *E. coli* to produce *n*-butanol at the level of grams per liter, which is competitive with the productivity of the industrial producer, *Clostridium acetobutylicum* [19–21]. However, the tolerance of *E. coli* to *n*-butanol limited further increase of the titer [22]. To address this issue, the strain SMB07/pML/pTL16 equipped with the SIM module was subjected to adaptive evolution under the stress of *n*-butanol (Fig. 4a).

The strain was turned on to the mutagenic state by the addition of IPTG in the mid-log phase. After 18–24 h of IPTG induction, cells were spread onto LB plates containing an inhibitory concentration of *n*-butanol and then incubated in a sealed container for 4–10 days to allow SIM to occur. It was confirmed that cells could survive for 9 days on lysogeny broth (LB) agar plates containing 10–12 g/L *n*-butanol (Additional file 1: Figure S4A). Therefore, SIM could occur in these stressed but viable cells. As expected, several colonies were seen on the *n*-butanol plates after 4 days of incubation and new colonies continued to appear after 7–10 days (Additional file 1: Figure S4B). The colonies were separately inoculated into LB medium containing aTc to switch to the high-fidelity state for evaluating their *n*-butanol tolerance. The best-performing colonies were subjected to the second round of adaptive evolution initiated by switching to the mutagenic state via IPTG addition. As shown in Fig. 4b, two rounds of adaptive evolution increased the *n*-butanol minimum inhibitory concentration (MIC) of SMB07/pML/pTL16 from 8 to 10 g/L, while another eight rounds further increased the MIC value to 12.5 g/L. In contrast, the control strain without the SIM module, FC40/pACYC184/pTAD, which underwent the same scheme, only reached an MIC of 10 g/L after ten rounds of evolution. In our previous work, the *mutL*-defective strain SMB07 improved the *n*-butanol MIC value of the parent strain from 9.5 to 13 g/L within 12 rounds of evolution [14]. Although the final *n*-butanol MIC obtained by the SIM module was a little lower than that obtained by SMB07, the evolution efficiency (i.e*.*, average increment per cycle) of the SIM module was higher.

The evolved strain, SMB705/pML/pTL16, exhibited superior *n*-butanol tolerance compared with its parent strain, SMB07/pML/pTL16 (Fig. 4c). It also showed higher osmotic tolerance than the parent strain in 50 and 60 g/L NaCl (Fig. 4d), similar acid tolerance at pH 4.8 (Fig. 4e) and similar growth capability in M9 minimal medium (Fig. 4f). Moreover, the evolved strain was genetically stable, with unaltered traits after 40 successive passages in LB medium.

## Discussion

The main goal of synthetic biology is to understand, design, and construct novel biological components to rewire organisms or even to create a new life [4]. Over a decade after the development of the first synthetic gene networks in 2000 (for example, the genetic toggle switch [15] and the repressilator [23]), several cellular physiological responses have been reconstructed by synthetic gene circuits, including cell-cell communication [24], a warning system of DNA damage [25], sensing the intensity of light [26], and taking up DNA from the environment [27]. However, adaptive evolution, which is an important cellular physiological response to stress [17, 28–30], has received little attention. In this study, we designed a module based on the SIM theory to mimic cellular responses of mutagenic DNA repair under stress. The module was constructed by placing genes related to up-regulation and down-regulation responses during SIM under the control of a toggle switch to regulate their expression/depression in a bistable way. *E. coli* cells transformed with the synthetic SIM module underwent controllable transition between a high-fidelity state and a mutagenic state upon addition of different inducers. The accelerated improvement of *n*-butanol tolerance of *E. coli* containing this module indicated that the complex cellular physiological response—adaptive evolution—could be simulated by the artificial synthetic module.

SIM has been considered with respect to microbial pathogenesis and antibiotic resistance, and tumor progression and chemotherapy resistance [10]. Recently, Rosenberg’s group has elucidated a molecular mechanism of SIM-mutagenic repair of DNA breaks [13, 31, 32]: the formation and repair of DNA double-strand breaks (DSBs) activate SOS response, which up-regulates Pol IV DNA polymerase. Then, a second stress, unrelated to the DSB, activates RopS, which recruits error-prone Pol IV, II, V, and/or I to participate in break repair, instead of/in addition to the use of high-fidelity Pol III. More than 93 genes are involved in the network of SIM, and most of them promote SIM by sensing stress and activating the key responses of RopS, RopE, and SOS [13]. However, the effects of individual genes on the SIM rates are unknown. Among the genes tested in this study, the SIM decelerator gene, *mutL*, seemed to play an essential role in SIM. Deletion of *mutL* increased the SIM rate by 82-fold, while the highest increase of SIM rate by single expression of any of the six SIM accelerator genes (*recA*, *dinB*, *umuD’*, *ropS*, *ropE*, and *nusA*) was only 14-fold. This result corresponds well with the observation that MMR genes in natural bacteria isolated from stressful environments are often deficient or show high sequence mosaicism [33–35]. These indicate that the recurrent loss and reacquisition of MMR genes might be a mechanism of natural adaptive evolution [35].

Surprisingly, expression of the SIM accelerator genes which were up-regulated during SIM did not always give positive effects on the SIM rate. These include the *umuD’* gene in the SOS response for error-prone DNA replication [36], the sigma factor *ropE* in heat-shock and envelope responses, and the transcription elongation factor *nusA* which recruits Pol IV (*dinB*) [12]. We suggest that these genes are not limiting factors in inducing SIM or have no function in the frameshift mutation of Lac^+^ reversion like *umuD’* [37]. For the other three genes tested, expression of *recA* was more efficient in elevating the SIM rate, with a 14-fold increase. The multifunctional RecA protein is responsible for DNA homologous recombination and activation of the SOS response and Pol V [38]. The important role of RecA in SIM has been demonstrated by the fact that deficiency of RecA-dependent recombination activity completely inhibited the Lac^*+*^ reversion mutation [17]. The translesion synthesis DNA polymerase Pol IV (*dinB*) and the principle sigma transcription factor RopS (*ropS*) in the general stress response are also essential for SIM. Up-regulation of the error-prone DNA polymerase Pol IV (*dinB*) can tilt its competition with DNA polymerases Pol I, II, and III, thus producing stress-induced mutations [37]. Previous studies have shown that deletion of *dinB* or *ropS* decreased the Lac^*+*^ reversion mutation rate by 80–90 % [39–42]. In this study, separate expression of the two genes increased the SIM rate by 4.5- to 5.7-fold. In addition, the increase of SIM rate by genes *recA*, *dinB*, and *ropS* was synergistic, as seen by the 32-fold increased SIM rate after expression of all three genes.

Evolutionary engineering composed of mutagenesis followed by selection has been widely applied to improve microbial tolerance to environmental stresses. Although mutagenesis can be easily turned on and off by addition of foreign physical or chemical mutagens, the use of a SIM module in this study enables in vivo generation of genome-wide mutations in non-dividing cells and thus synchronizes the mutagenesis and selection process in a single-plate incubation step. Moreover, the SIM module avoids the use of any toxic mutagen to trigger the mutagenic state.

In comparison with our previous work which used the *mutL*-defective strain to perform the SIM-based adaptive evolution, the most important advantage of using the SIM module is that it offers the feasibility of switching the mutagenic state back to the high-fidelity stage to stably maintain the improved phenotype at the end of evolution. However, the *mutL* gene is permanently deleted in SMB07 so that the strain is always in its mutagenic state. Cultivation of such a hypermutable strain will be problematic because mutations continue to emerge across the genome and the desired traits might disappear during long-term passages. Moreover, the SIM module expands the dynamic range of the SIM rates and provides the possibility of their fine-tuning. The strain SMB07 has a lower and fixed (82-fold increased) SIM rate, while the SIM module can reach a much higher (2923-fold increased) SIM rate. Different fluorescence values were generated by the toggle switch upon addition of different concentrations of IPTG or aTc (Additional file 1: Figure S1C and D), providing the possibility of artificial regulation of the SIM rates by varying the inducer concentrations. It has been reported that adaption to a specific stress prefers a specific mutagenesis strength [22]. Therefore, such a fine-tuning of the SIM rates will be beneficial for applying this method in improving microbial tolerance against various stresses.

## Conclusion

In this study, a synthetic module based on the SIM theory was developed to stimulate cellular up- and down-regulation responses during SIM. Operation of this module in *E. coli* simply by adding different inducers enables cellular transition between high-fidelity and mutagenic states. Such a transition then triggers microbial autonomous adaptive evolution under stressful conditions. This work provides a useful SIM module and a feasible approach to accelerate microbial adaptive evolution for strain engineering. Moreover, it also advances our understanding of the molecular mechanisms of SIM and natural adaptive evolution, which might provide insight into microbial pathogenesis and antibiotic resistance.

## Methods

### Strains and culture conditions

The *E. coli* strains and plasmids used in this study are shown in Additional file 1: Table S1. Bacteria were grown in LB medium or M9-glycerin minimal medium (M9 medium supplemented with 20 μg/mL thiamine, 0.001 % gelatin, and 0.2 % glycerin). Ampicillin (100 μg/mL) and tetracycline (25 μg/mL) were added when necessary.

### Plasmid construction

The sequences of promoters, terminators, ribosomal binding sites, and primers are summarized in Additional file 1: Table S2. Two basic BioBrick units, P_L_tetO-1-*lacI*-T1 and Ptrc-2-*tetR*-T2, were first constructed by fusion PCR and then cloned into pUC19, yielding pLacI and pTetR, respectively (Additional file 1: Figure S2A). Another seven gene units were constructed by replacing *lacI* and/or *tetR* between *Sal*I/*Xho*I sites with *gfp*, *dinB*, *recA*, *ropS*, *ropE*, *nusA*, *umuD’*, or *mutL*, yielding pTrcGFP, pDinB, pRecA, pRpoS, pRpoE, pNusA, pUmuD, pTetGFP, and pMutL (Additional file 1: Figure S2A). pTL01 was constructed by integration of pLacI and pTetR (Additional file 1: Figure S2C). The gene units of *recA*, *dinB*, *umuD’*, *ropS*, *ropE*, or *nusA* were inserted and integrated into pTL01 using standard BioBrick assembly (15), yielding pTL02 to pTL16 (Fig. 2). The *mutL* gene unit was cloned into *Drd*I/*Ahd*I sites of pACYC184, yielding pML (Additional file 1: Figure S2D).

### Lac^+^ reversion mutation assay

The SIM rate of *E. coli* FC40 harboring pTL01-16 was determined by the Lac^*+*^ reversion mutation assay described previously [14] with slight modifications. aTc was added to liquid M9-glycerin medium at a concentration of 500 ng/mL to stringently repress gene expression before plating. Appropriate antibiotics were added to the medium for the maintenance of plasmids when necessary. *E. coli* FC29/pTAD was used for scavenger cells.

### SIM-based adaptive evolution for improving *n*-butanol tolerance

Similar to a previously reported method [14], *E. coli* SMB07/pML/pTL16 was cultured in LB broth with 25 μg/mL tetracycline and 100 μg/mL ampicillin for 5–6 h. Then, 100 μM IPTG and a sub-inhibitory concentration of *n*-butanol (6 g/L for the initial cycle) were added, and the incubation continued for 18–24 h. A 200-μL aliquot of culture was spread onto freshly prepared LB agar plates containing 25 μg/mL tetracycline, 100 μg/mL ampicillin, and an inhibitory concentration of *n*-butanol (9–12 g/L for the initial cycle). These plates were incubated at 37 °C for 4–10 days in a sealed container. The colonies that gradually appeared were streaked on an LB plate with 25 μg/mL tetracycline, 100 μg/mL ampicillin, the same concentration of *n*-butanol, and 500 ng/mL aTc. After 2 days of incubation, the colonies exhibiting a rapid growth rate were selected for comparative analysis of growth capability in a sealed tube of LB medium containing 25 μg/mL tetracycline, 100 μg/mL ampicillin, the same concentration of *n*-butanol, and 500 ng/mL aTc. The best-performing strains were selected for the next cycle of adaptive evolution.

### Minimum inhibitory concentration assay of *n*-butanol

A fresh single colony was inoculated into 10 mL LB broth and cultured for 6–8 h at 37 °C, 200 rpm. Fresh LB medium in a sealed tube was then inoculated with a 1:50 aliquot of the culture and different concentrations of *n*-butanol added. The OD_600_ value of cultures was detected using 96-well plates in a microtiter plate reader (SpectraMAX 190, Molecular Devices, USA). The concentration of *n*-butanol that restricted the increase of OD_600_ by twofold or less was defined as the MIC.

## Additional file

Additional file 1: **Supplementary material.** **Table S1.** *E. coli* strains and plasmids used in this study. **Table S2.** Sequences used in this study. **Table S3.** The Lac^+^ colonies of strains FC40 containing different plasmids on M9-lactose plates and their calculated SIM rates. **Table S4.** The Lac^+^ colonies of different strains on M9-lactose plates and their calculated SIM rates. **Figure S1.** Bistable control of the synthetic toggle switch using *gfp* as a reporter. **Figure S2.** Structures of the main plasmids and the induction strength of three ribosomal binding site sequences. **Figure S3.** The effect of different inducers on *gfp* expression in strain SMB07/pML/pTLCG. **Figure S4.** *E. coli* cells spread on LB agar plates with an inhibitory concentration of *n*-butanol can survive for 9 days and gradually formed colonies over 4–10 days.

## Abbreviations

aTc: anhydrotetracycline
DSB: double-strand break
GFP: green fluorescent protein
IPTG: isopropyl-β-D-1-thiogalactopyranoside
MIC: minimum inhibitory concentration
MMR: mismatch repair
SIM: stress-induced mutagenesis
