# Supplementary material for: Development of a stress-induced mutagenesis module for autonomous adaptive evolution of Escherichia coli to improve its stress tolerance
Source: Biotechnol Biofuels. 2015 Jun 26;8:93. doi: 10.1186/s13068-015-0276-1 (PMC4487801; doi:10.1186/s13068-015-0276-1)
Supplement: Supplementary file 1 — Supplementary material. Table S1. E. coli strains and plasmids used in this study. Table S2. Sequences used in this study. Table S3. The Lac+ colonies of strains FC40 containing different plasmids on M9-lactose plates and their calculated SIM rates. Table S4. The Lac+ colonies of different strains on M9-lactose plates and their calculated SIM rates. Figure S1. Bistable control of the synthetic toggle switch using gfp as a reporter. Figure S2. Structures of the main plasmids and the induction strength of three ribosomal binding site sequences. Figure S3. The effect of different inducers on gfp expression in strain SMB07/pML/pTLCG. Figure S4. E. coli cells spread on LB agar plates with an inhibitory concentration of n-butanol can survive for 9 days and gradually formed colonies over 4–10 days. [file 13068_2015_276_MOESM1_ESM.docx]

**Supplementary material**

**Development of a stress-induced mutagenesis module for autonomous adaptive evolution of *Escherichia coli* to improve its stress tolerance**

Linjiang Zhu, Yin Li, Zhen Cai

**Table S1 *E. coli* strains and plasmids used in this study**

| Strain/Plasmid | Genotype | Source |
| --- | --- | --- |
| *E. coli* JM109 | *recA1 mcrB^+^ hsdR17* | Lab storage |
| *E. coli* FC29 | Rif^S^, F′ Δ(*lacIlacZ*), *ara*Δ(*lacproB*) *thi* | (16) |
| *E. coli* FC40 | Rif^R^, F′ (*lacIΩZ^**^*), *ara*Δ(*lacproB*) *thi* | (16) |
| *E. coli* SMB07 | a *mutL*-deletedmutant strain of *E. coli* FC40 | (13) |
| pUC19 | *ori*ColE1, Amp^r^, | Lab storage |
| pTAK117 | *ori*ColE1, Amp^r^,contained a genetic toggle switch composed of two promoters (P_L_s1con and Ptrc-2) and two repressors (CIts and LacI) | (14) |
| pIKE107 | *ori*ColE1, Amp^r^, contained a genetic toggle switch composed of two promoters (P_L_tetO-1 and Ptrc-2) and two repressors (TetR and LacI) | (14) |
| pKD46 | *ori*R101,*repA*101(*ts*),*araBp*2*gam*2*bet*2*exo*,Amp^r^ | Lab storage |
| pKD4 | [*ori*R6K](http://cgsc.biology.yale.edu:80/cgi-bin/sybgw/cgsc/Mutation/64912),[*bla*(Amp)](http://cgsc.biology.yale.edu:80/cgi-bin/sybgw/cgsc/Mutation/71112) ,[*rgnB*(Ter)](http://cgsc.biology.yale.edu:80/cgi-bin/sybgw/cgsc/Mutation/71730),[Kan](http://cgsc.biology.yale.edu:80/cgi-bin/sybgw/cgsc/Mutation/71790)^r^ | Lab storage |
| pCP20 | *Ts-rep*,Cm^r^, Amp^r^ | Lab storage |
| pACYC184 | *ori*p15A, Tc^r^,Cm^r^ | Lab storage |
| pTAD | Derivation from pTAK117,*PstI*digestedpTAK117 to delete the parts of *lacI, clts,* P_L_s1con*,* andPtrc-2 in the toggle switch. | This study |
| pML | Derivation from pACYC184, expressing *mutL* gene underP_L_tetO-1 | This study |
| pLacI | BioBrickunit derived from pUC19,P_L_tetO-1, rbs_1_, *lacI*, T_1_ terminator | This study |
| pTetR | BioBrickunit derived from pUC19, Ptrc-2, rbs_1_, *tetR*, T_2_ terminator | This study |
| pMutL | BioBrick unit derived from pUC19, P_L_tetO-1, rbs_1_, *mutL*, T_1_ terminator | This study |
| pRecA | BioBrick unit derived from pUC19, Ptrc-2, rbs_1_, *recA*, T_2_ terminator | This study |
| pDinB | BioBrick unit derived from pUC19, Ptrc-2, rbs_3_, *dinB*, T_2_ terminator | This study |
| pUmuD | BioBrick unit derived from pUC19, Ptrc-2, rbs_1_, *umuD′*, T_2_ terminator | This study |
| pRpoS | BioBrick unit derived from pUC19, Ptrc-2, rbs_1_, *rpoS*, T_2_ terminator | This study |
| pRpoE | BioBrick unit derived from pUC19, Ptrc-2, rbs_2_, *rpoE*, T_2_ terminator | This study |
| pNusA | BioBrickunit derived from pUC19, Ptrc-2, rbs_2_, *nusA*, T_2_terminator | This study |
| pTCG-01/02/03 | BioBrickunit derived from pUC19, Ptrc-2, rbs_1/2/3_, *gfp*, T_2_ terminator | This study |
| pTEG | BioBrickunit derived from pUC19, P_L_tetO-1, rbs_1_, *gfp*, T_1_ terminator | This study |
| pTL01 | *ori*ColE1, Amp^r^, contained a genetic toggle switch composed of two promoter (P_L_tetO-1 and Ptrc-2) and two repressor (TetR and LacI), constructed by assembly of pLacI and pTetR | This study |
| pTL02 | Derivation from pTL01,expressing *dinB* | This study |
| pTL03 | Derivation from pTL01, expressing *recA* | This study |
| pTL04 | Derivation from pTL01, expressing*rpoS* | This study |
| pTL05 | Derivation from pTL01, expressing *rpoE* | This study |
| pTL06 | Derivation from pTL01, expressing*nusA* | This study |
| pTL07 | Derivation from pTL01, expressing*umuD′* | This study |
| pTL08 | Derivation from pTL01, expressing*dinB* and*recA* | This study |
| pTL09 | Derivation from pTL01, expressing *dinB* and*nusA* | This study |
| pTL10 | Derivation from pTL01, expressing *dinB* and*rpoS* | This study |
| pTL11 | Derivation from pTL01, expressing *recA* and*nusA* | This study |
| pTL12 | Derivation from pTL01, expressing *recA* and*rpoS* | This study |
| pTL13 | Derivation from pTL01, expressing *rpoS* and*nusA* | This study |
| pTL14 | Derivation from pTL01, expressing *recA*,*dinB* and*rpoS* | This study |
| pTL15 | Derivation from pTL01, expressing *dinB*,*rpoS* and*recA* | This study |
| pTL16 | Derivation from pTL01, expressing *rpoS*,*dinB* and*recA* | This study |
| pTLCG | Derivation from pTL01, expressing *gfp* | This study |
| pTLEG | Derivation from pTL01, expressing *gfp* | This study |

**Table S2 Sequences used in this study**

| Name | Sequence (restriction sites underlined) | Note |
| --- | --- | --- |
| **Promoter** |  |  |
| Ptrc-2 | ACCATCGAATGGCTGAAATGAGCTGTTGACAATTAATCATCCGGCTCGTATAATGTGTGGAATTGTGAGCGGATAACAATTTCACACAGGAAACCGGT |  |
| P_L_tetO-1 | AACGTAAATGCTCCCTATCAGTGATAGAGATTGACATCCCTATCAGTGATAGAGATACTGAGCACATCAGCAGGACGCACTGACCA |  |
| **Terminator** |  |  |
| T_1_ | GTTGTTACCTCGTTACCTTTGGTCGAAAAAAAAAGCCCGCACTGTCAGGTGCGGGCTTTTTTCTGTGTTTCCTG |  |
| T_2_ | ATGACACTGTGATCTAAAAAGAGCGACTTCGGTCGCTCTTTTTTTTACCTGATAAAATGAAGTTAAAGGA CT |  |
| **Ribosomal binding site** |  |  |
| RBS_1_ | TA**AGGAGG**AAGAAAC**ATG** |  |
| RBS_2_ | **AGGA**CGGTTCG**ATG** |  |
| RBS_3_ | **AGGA**AACGGTTCG**ATG** |  |
| **Primer** |  |  |
| Ptet-F | cttgac*GCATGCGCTAGCACTAGT*AACGTAAATGCTCCCTATCAGTGATAGAGATTGA | Amplify P_L_tetO-1 |
| Ptet-rbs_1_-R | TCCTAGTCGACCATTGTTTCCTCCTCTCAATCGCTCAATGTGGTCAGTGCGTCCTGCTG |  |
| LacI-F | AATCCTA*GTCGAC*AAACCAGTAACGTTATACGATGTCG | Construction of LacI-T_1_by fusion PCR |
| LacI-T-R | TCTGTGTTTCCTG*CTCGAG*TCACTGCCCGCTTTCCAGTCGGGA |  |
| T1-F | CAGTGA*CTCGAG*caggaaacacagaaaaaagcccgcacc |  |
| T1-R | AGATCA*GGATCCTCTAGA*GTTGTTACCTCGTTACCTTTGGTCG |  |
| Linker(T1) | CACAGAAAAAAGCCCGCACCTGACAGTGCGGGCTTTTTTTTTCGACCAAAGGTAACGAG |  |
| Ptrc-F | cacatctc*GGATCCTCTAGA*ccatcgaatggctgaaatgagctg | AmplifyPtrc-2 |
| Ptrc-rbs_1_-R | gtcaa*GTCGAC*CATGTTTCTTCCTCCTTATACCGGTTTCCTGTGTGAAATTGTTATCCG |  |
| Ptrc-rbs_2_-R | cgtcaaGTCGACCATCGAACCGTCCTTATACCGGTTTCCTGTGTGA |  |
| Ptrc-rbs_3_-R | cgtcaaGTCGACCATCGAACCGTTTCCTTATACCGGTTTCCTGTGTGA |  |
| TetR-F | ccgtcaa*GTCGAC*agattagataaaagtaaagtgatta | Construction of TerR-T_2_ by fusion PCR |
| TetR-R | TAGATCACAGTGTCAT*CTCGAG*TTAAGACCCACTTTCACATTTAAGTTG |  |
| T2-F | GTCTTAA*CTCGAG*atgacactgtgatctaaaaagagcg |  |
| T2-R | TAGATCA*GCATGCGCTAGCACTAGT*CAGTCCTTTAACTTCATTTTATCAGG |  |
| Linker(T2) | CTGTGATCTAAAAAGAGCGACTTCGGTCGCTCTTTTTTTTACCTGATAAAATGAAGTTA |  |
| GFP-Xho-R | aatgcg*CTCGAG*TTATTTGTATAGTTCATCCATGC | Amplify*gfp* |
| GFP-Sal-F | tggcaa*GTCGAC*agtaaaggagaagaacttttcactg |  |
| DinB-Sal-F | tggcaa*GTCGAC*CGTAAAATCATTCATGTGGATATG | Amplify*dinB* |
| DinB-Xho-R | aatgcg*CTCGAG*TCATAATCCCAGCACCAGTTGTC |  |
| RpoS-Sal-F | tggcaa*GTCGAC*AGTCAGAATACGCTGAAAGTTCATG | Amplify*rpoS* |
| RpoS-Xho-R | aatgcg*CTCGAG*TTACTCGCGGAACAGCGCTTCG |  |
| RecA-Sal-F | tggcaa*GTCGAC*GCTATCGACGAAAACAAACAGA | Amplify*recA* |
| RecA-Xho-R | aatgcg*CTCGAG*TTAAAAATCTTCGTTAGTTTCTGC |  |
| RpoE-Sal-F | tggcaa*GTCGAC*AGCGAGCAGTTAACGGACCAGGTC | Amplify*rpoE* |
| RpoE-Xho-R | aatgcg*CTCGAG*TCAACGCCTGATAAGCGGTTGAAC |  |
| MutL-Sal-F | tggcaa*GTCGAC*CCAATTCAGGTCTTACCGCCAC | Amplify*mutL* |
| MutL-Xho-R | aatgcgCTCGAGTCACTCATCTTTCAGGGCTTTTATCG |  |
| NusA-Sal-F | tggcaa*GTCGAC*AACAAAGAAATTTTGGCTGTAGT | Amplify*nusA* |
| NusA-Xho-R | aatgcg*CTCGAG*TTACGCTTCGTCACCGAACCAGC |  |
| UmuD-Sal-F | tggcaa*GTCGAC*GGCTTTCCTTCACCGGCAGCAG | Amplify *umuD*′ |
| UmuD-XhoR | aatgcg*CTCGAG*TCAGCGCATCGCCTTAACGACGTGG |  |
| pUC-F | gcgattaagttgggtaacgccag | Universal primer |
| pUC-R | TTCACACAGGAAACAGCTATGAC |  |
| Drd-pUC-F | ccttga*GACCCGGTCGTC*gcgattaagttgggtaacgccag |  |
| Ahd-M13-R | ATCTT*gactgagagtc*TTCACACAGGAAACAGCTATGAC |  |
| mutL-koF | CTCGATGCAGGTGCGACGCGTATCGATATTGATATCGAAC**TGTAGGCTGGAGCTGCTTC** | Amplify*kan* for *mutL*deletion |
| mutL-koR | TGGCCTGTGCCATTGACCACTGCGCATGTTCGCTCATCAG**TGGGAATTAGCCATGGTCC** |  |
| Kan-R | CGGCCACAGTCGATGAATCC |  |

**Table S3 The Lac^+^ colonies of strains FC40 containing different plasmids on M9-lactose plates and their calculated SIM rates**

| Plasmid | Lac^+^ colonies per 10^6^ cells ^a^ | | | | | SIM rate (Lac^+^ colonies per 10^6^ cells per day) ^b^ |
| --- | --- | --- | --- | --- | --- | --- |
|  | 2 d | 3 d | 4 d | 5 d | 6 d |  |
| pTL01 | 8.8±2.8 | 20±5 | 29±6 | 38±5 | 45±8 | 9.1±0.7 |
| pTL02 | 21.3±10 | 75±15 | 133±35 | 153±48 | 185±60 | 40.5±4.4 |
| pTL03 | 90±32 | 203.3±87 | 325±98 | 495±102 | 585±120 | 123.8±9.5 |
| pTL04 | 31.4±15 | 94.7±28 | 154±55 | 198±65 | 239±90 | 52.0±6.6 |
| pTL05 | 1.6±0.8 | 3.5±1.5 | 6.9±3.2 | 10.8±4.5 | 12±5 | 2.6±0.4 |
| pTL06 | 11.7±2.6 | 26.6±5 | 39.8±8 | 49±15 | 58.1±19 | 11.6±1.4 |
| pTL07 | 4.6±0.6 | 13.4±2.4 | 20.2±3.6 | 28.2±3.7 | 34.2±4.5 | 7.4±0.3 |
| pTL08 | 106.7±25 | 254±80 | 405±90 | 586±100 | 1030±210 | 231.0±14.7 |
| pTL09 | 20±8 | 65±25 | 92±24 | 117±32 | 130±62 | 27.5±4.4 |
| pTL10 | 27±15 | 84±28 | 139±36 | 164±43 | 192±52 | 41.2±4.2 |
| pTL11 | 20±5 | 62±21 | 120±35 | 140±46 | 152±50 | 33.1±3.4 |
| pTL12 | 37±11 | 112±23 | 192±37 | 227±52 | 262±65 | 56.3±4.8 |
| pTL13 | 11±5 | 40±12 | 68±18 | 81±17 | 102±32 | 22.8±2.3 |
| pTL14 | 32±5 | 102±12 | 171±16 | 262±32 | 413±45 | 95.3±3.1 |
| pTL15 | 34±6 | 93±11 | 173±20 | 285±35 | 336±48 | 75.5±3.4 |
| pTL16 | 225±50 | 576±120 | 786±182 | 1069±218 | 1380±203 | 288.8±15.6 |

^a^Mean value ± standard deviations of three independent experiments

^b^SIM rate was calculated by (The value at Day 6- The value at Day 2)/4

**Table S4 The Lac^+^ colonies of different strains on M9-lactose plates and their calculated SIM rates**

| Inducer | Strain | Lac^+^ colonies per 10^6^ cells ^a^ | | | | | SIM rate (Lac^+^ colonies per 10^6^ cells per day) ^b^ |
| --- | --- | --- | --- | --- | --- | --- | --- |
|  |  | 2 d | 3 d | 4 d | 5 d | 6 d |  |
| IPTG | FC40 | 0.13±0.05 | 0.3±0.12 | 0.8±0.26 | 1.3±0.39 | 2±0.6 | 0.5±0.04 |
|  | SMB07 | 29±9 | 62±16 | 95±15 | 122±21 | 182±26 | 38.3±2.2 |
|  | FC40/pTL01 | 8.8±2.8 | 20±5 | 29±6 | 38±5 | 45±8 | 9.1±0.7 |
|  | FC40/pTL16 | 225±50 | 576±120 | 786±182 | 1069±218 | 1380±203 | 288.8±5.8 |
|  | SMB07/pML/pTL16 | 367±92 | 2838±502 | 4200±321 | 5333±412 | 5833±356 | 1366.7±28 |
| aTc | FC40 | 0.09±0.04 | 0.21 ±0.1 | 0.65 ±0.13 | 1.1 ±0.18 | 1.6±0.27 | 0.4±0.08 |
|  | SMB07/pML | 0.3 ±0.09 | 3.2 ±0.7 | 10 ± 1.8 | 12.8±2.1 | 14.5±2.1 | 3.6±0.6 |

^a^Mean value ± standard deviations of three independent experiments

^b^SIM rate was calculated by (The value at Day 6 - The value at Day 2)/4


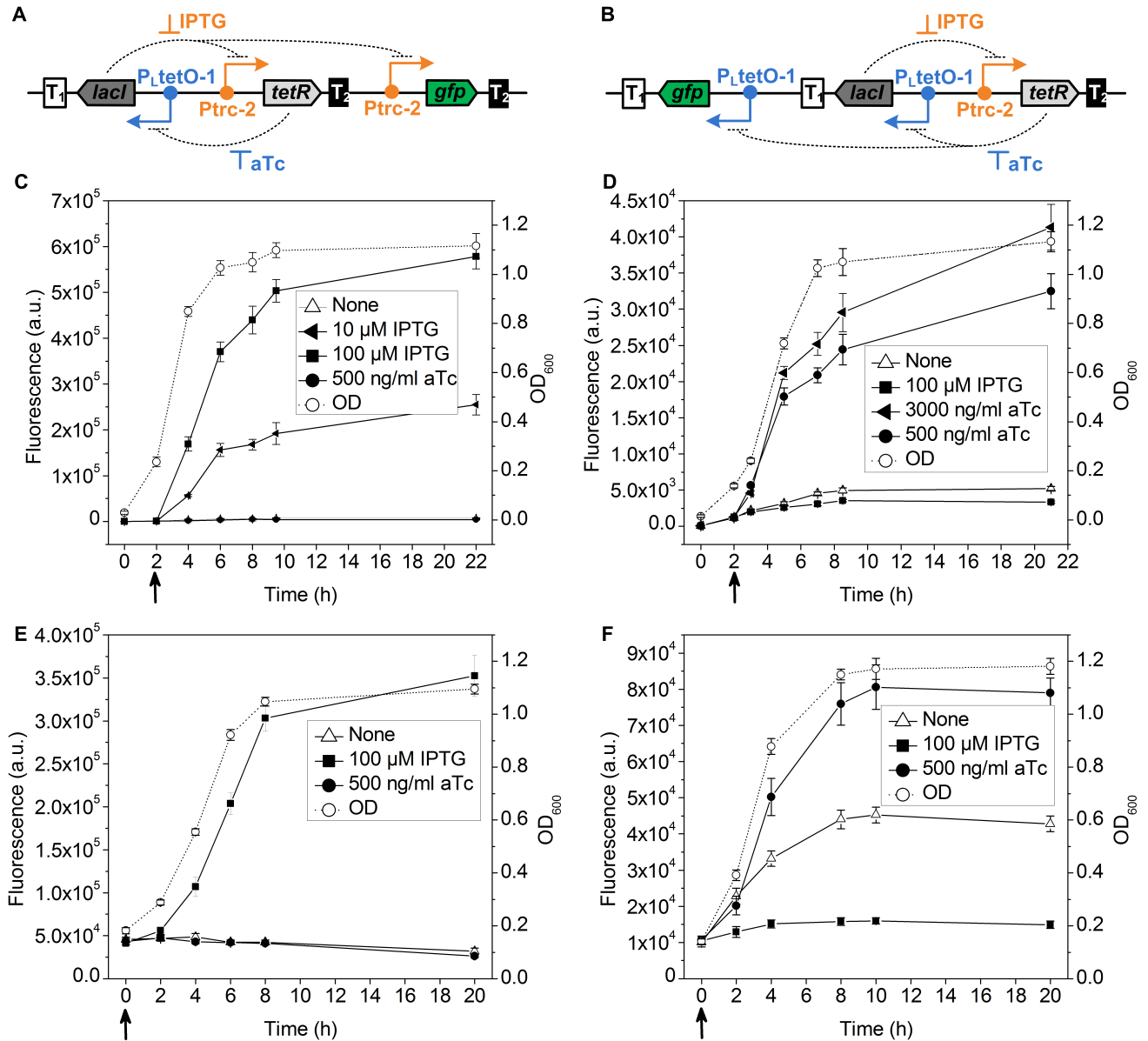


**Figure S1 Bistable control of the synthetic toggle switch using *gfp* as a reporter.** The *gfp* gene was separately placed under the control of Ptrc-2 or P_L_tetO-1 in pTL01, yielding plasmids pTLCG (**A**) and pTLEG (**B**). *E. coli* FC40 harboring pTLCG (**C**) or pTLEG (**D**) were cultivated overnight at 37°C, 200 rpm. Then, 10 mL LB broth was inoculated with 200 μL culture, and incubated with shaking at 37°C for 2 h. When the cells reached the mid-exponential phase (OD=0.3 in a 96-well microplate), 10 or 100 μM IPTG, 500 or 3000 ng/mL aTc, or nothing was added to the medium (indicated by arrows). The GFP fluorescence in 200 μL of culture in a black 96-well plate was recorded by Infinite M200 microplate reader (TECAN, Switzerland). OD_600_ values of the same culture were determined in a 96-well transparent plate by Spectra MAX 190 microplate reader (Molecular Devices, USA). To test whether the toggle switch still worked when the *gfp* gene was already expressed, the overnight cultures were first inoculated into media containing 100 μM IPTG (FC40/pTLCG) (**E**) or 500 ng/mL aTc (FC40/pTLEG) (**F**), and then induced for 12 h at 37°C. Then, 1 mL of the pre-induced culture was collected, washed with fresh LB broth, and inoculated into 10 mL LB broth. After that, 100 μM IPTG, or 500 ng/mL aTc or nothing was added as indicated by arrows. The GFP fluorescence and OD_600_ values were determined as above. Ampicillin was added to all media at a final concentration of 100 μg/mL. Three independent cultures were prepared for each condition.





**Figure S2** **Structures of the main plasmids and the induction strength of three ribosomal binding site (RBS) sequences.** **(A)** Eleven genes, each with their promoters, RBSs, and terminators, flanked by the modified BioBrick enzymatic connections (*Sph*I instead of *Pst*I), were cloned into plasmid pUC19. Restriction endonucleases: E: *Eco*RI; X: *Xba*I; Se: *Spe*I; Sh: *Sph*I. (**B**) *E. coli* FC40 transformed with pTrcGFP1/2/3, which contain three different RBS sequences (shown in Table S2) at the upstream of *gfp*, were cultured in LB broth to mid-logarithmic phase at 37°C, 200 rpm. At point zero, 0 μM (open symbols) or 50 μM IPTG (solid symbols) was added and GFP fluorescence was recorded versus time as described in Figure S1. Square, circle, and triangle symbols represent RBS_1_, RBS_2_, and RBS_3_, respectively. Ampicillin was added to all media at a final concentration of 100 μg/mL. The plasmid maps of pTL01 (**C**) and pML (**D**) are shown.


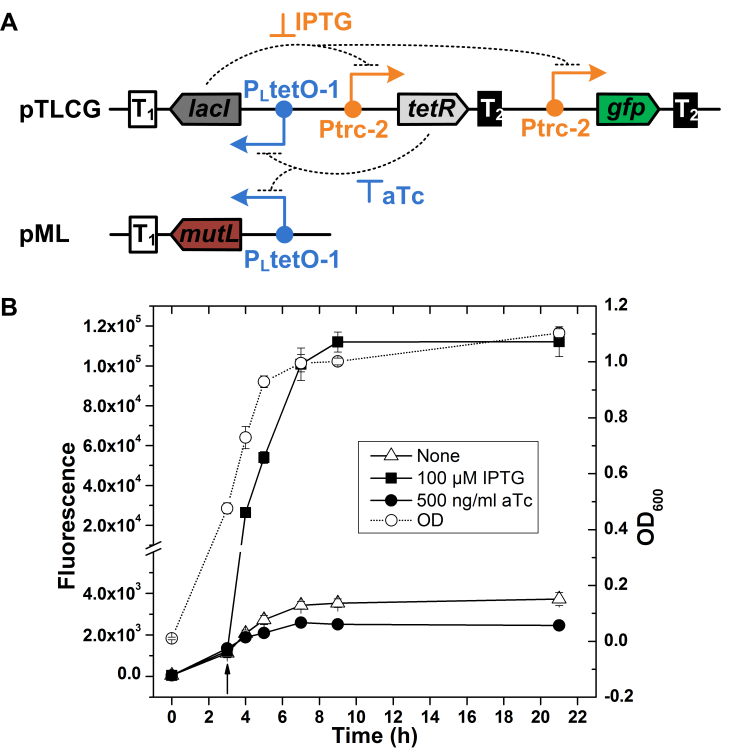


**Figure S3 The effect of different inducers on *gfp* expression in strain SMB07/pML/pTLCG.** The strain was cultivated overnight at 37°C and 1:50 diluted in LB broth containing 100 μg/mL of ampicillin and 25 μg/mL of tetracycline for another 3 h of shaking at 37°C. 100 μM IPTG, or 500 ng/mL aTc, or nothing was added to the medium as indicated by the arrows. The GFP fluorescence and OD_600_ values were determined as Figure S1. Three independent cultures were prepared for each condition.





**Figure S4** *E. coli* cells spread on LB agar plates with an inhibitory concentration of *n*-butanol can survive for 9 days and gradually formed colonies over 4–10 days. **(A)** Strain SMB07/pML/pTL16, pre-stressed by 6 g/L *n*-butanol for 18–20 h, was plated onto LB agar plates containing 9 g/L, 10 g/L, or 12 g/L *n*-butanol, and incubated in a sealed container at 37°C. On each day, a 28.3-mm^2^ area of agar was cut out using an Oxford cup and tweezers. The agar sample was mixed with 1 mL M9 salt solution in a 2-mL centrifugation tube and mixed by vortexing for 5 min at maximum speed. The obtained cell suspensions were spread on LB agar plates for colony-forming unit (CFU) counting. Three independent cell cultivations were performed for each strain and two parallel Oxford cup samples were taken from each plate. **(D)** Colonies on the LB-butanol agar plate (indicated by arrows) gradually appeared over 4–10 days of incubation in a sealed box.
